# A Minimal Load‐and‐Lock Ru^II^ Luminescent DNA Probe

**DOI:** 10.1002/anie.202108077

**Published:** 2021-08-11

**Authors:** Matthew D. Newton, Simon D. Fairbanks, Jim A. Thomas, David S. Rueda

**Affiliations:** ^1^ Department of Infectious Disease Faculty of Medicine Imperial College London London W12 0NN UK; ^2^ Single Molecule Imaging Group, MRC- London Institute of Medical Sciences London W12 0NN UK; ^3^ Department of Chemistry University of Sheffield Sheffield S3 7HF UK; ^4^ Department of Molecular Biology and Biotechnology University of Sheffield Western Bank Sheffield S10 2TN UK

**Keywords:** DNA imaging, optical tweezers, single molecule microscopy, threading intercalators

## Abstract

Threading intercalators bind DNA with high affinities. Here, we describe single‐molecule studies on a cell‐permeant luminescent dinuclear ruthenium(II) complex that has been previously shown to thread only into short, unstable duplex structures. Using optical tweezers and confocal microscopy, we show that this complex threads and locks into force‐extended duplex DNA in a two‐step mechanism. Detailed kinetic studies reveal that an individual stereoisomer of the complex exhibits the highest binding affinity reported for such a mono‐intercalator. This stereoisomer better preserves the biophysical properties of DNA than the widely used SYTOX Orange. Interestingly, threading into torsionally constrained DNA decreases dramatically, but is rescued on negatively supercoiled DNA. Given the “light‐switch” properties of this complex on binding DNA, it can be readily used as a long‐lived luminescent label for duplex or negatively supercoiled DNA through a unique “load‐and‐lock” protocol.

## Graphical abstract

Using single‐molecule optical tweezers and confocal microscopy we perform kinetic analysis of a novel dinuclear ruthenium(II) DNA imaging probe. Reversable binding is observed on relaxed DNA, however on stretching DNA, threading can occur, resulting in high affinity stable binding. Threading is highly stereoisomer specific and dependent on DNA supercoiling state. This DNA probe has exciting potential for DNA structure specific imaging.

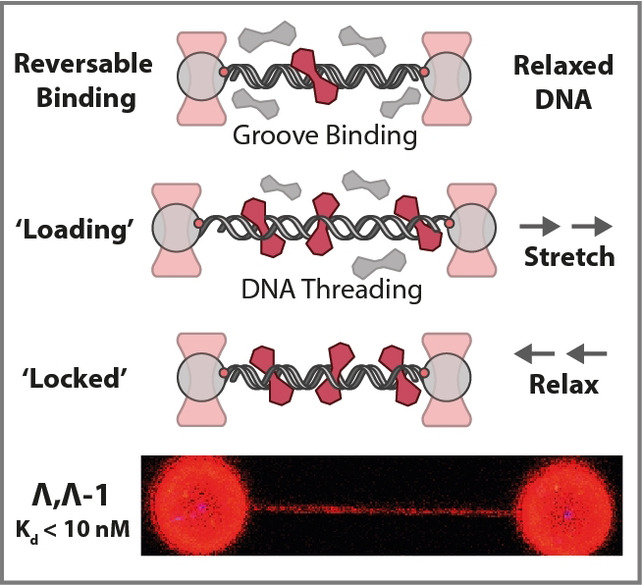

## Introduction

Owing to its central role in the process of life, new methods to visualize DNA and its dynamics are constantly being sought. The ability to image DNA, DNA‐protein interactions, and changes in DNA structure in vivo and in vitro has provided key insights into fundamental cellular function.^[1]^ In this context, fluorescence microscopy has proven to be particularly versatile and this has led to a large variety of luminescent small organic molecules being investigated and developed as DNA imaging probes.[^2^, ^3^] Due to some of the drawbacks of these conventional probes, the use of transition metal complexes in this role has been explored.

**Figure 1:**
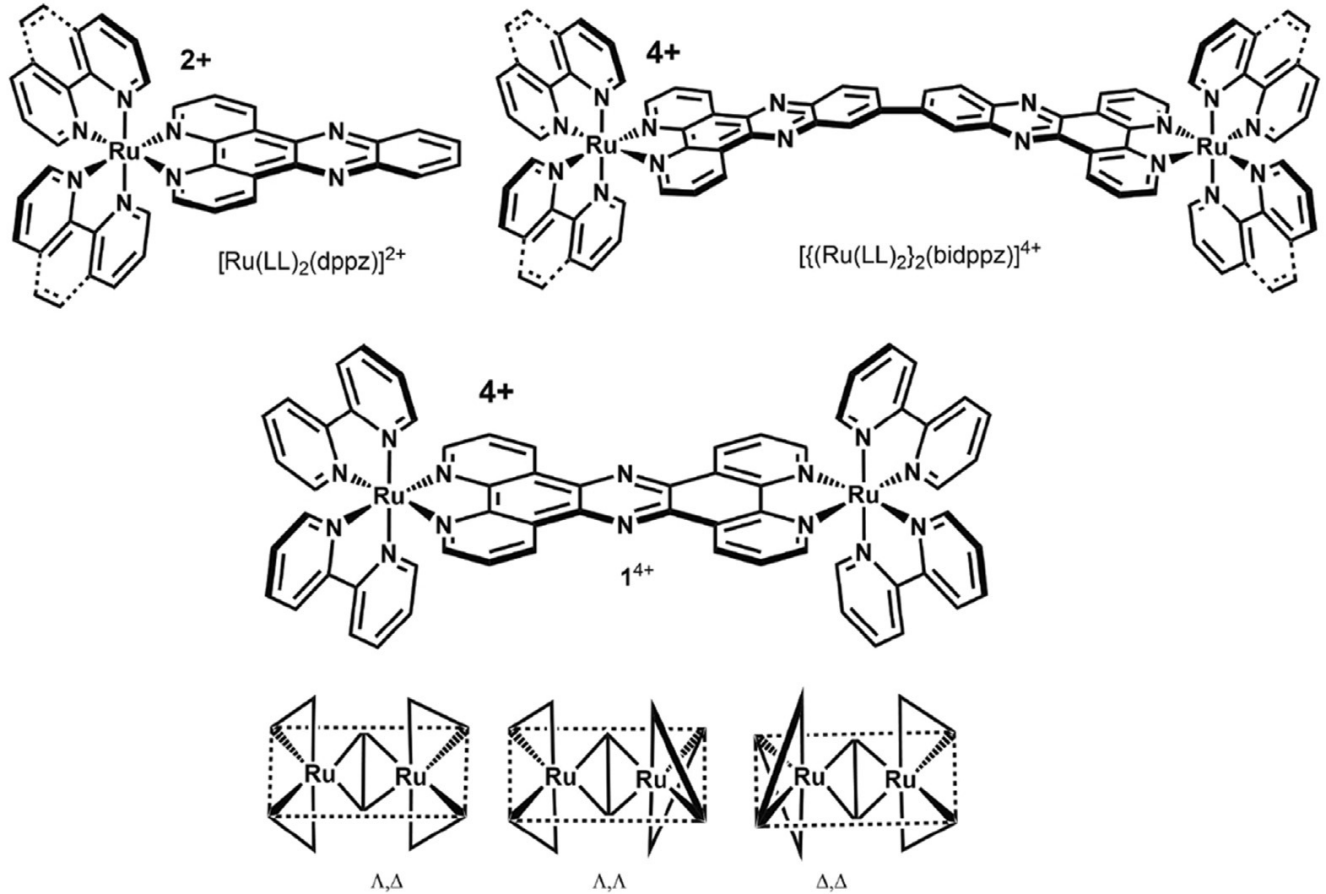
Chemical structures relevant to this study. Top: Chemical structure of DNA intercalator [Ru(LL)_2_(dppz)]^2+^ and an example of a related threading complex, [{(Ru(LL)_2_}_2_(bidppz)]^4+^, where LL=2,2′‐bipyridine (bpy) or 1,10‐phenanthroline (phen). Bottom: [{(Ru(bpy)_2_}_2_(tpphz)]^4+^ (**1** ^4+^) and representations of its three stereoisomers Λ,Δ, Λ,Λ and Δ,Δ.

The properties of water‐soluble salts of *d* ^6^ metal centres, especially polypyridyl Ru^II^ cations, have proven to be particularly promising; as these species typically exhibit photostable^[3]^ metal‐to‐ligand charge transfer (MLCT) excited states, they frequently possess bright long‐lived emission with large Stokes shifts.[^4^, ^5^, ^6^, ^7^, ^8^, ^9^, ^10^, ^11^, ^12^] Due to this attractive combination of properties, complexes with extended aromatic ligands capable of intercalating into DNA were investigated and key early studies revealed that [Ru^II^ (LL)_2_(dppz)]^2+^ (LL=2,2′‐bipyridine or 1,10‐phenanthroline, dppz=dipyrido[3,2‐a:2′,3′‐cj phenazine), Figure 1, displays a “DNA light switch” effect.^[13]^ Through hydrogen‐bonding interactions with the dppz ligand, the emissive state of this complex is solvent‐quenched until solvent shielding through DNA intercalation “switches on” luminescence.[^14^, ^15^, ^16^, ^17^] Consequently, a number of dinuclear ruthenium complexes based on linked Ru^II^(dppz) fragments have been developed that thread through DNA duplexes, see Figure 1 for examples.[^18^, ^19^] In this binding motif, one of the bulky ruthenium centres passes through the DNA duplex to produce a final structure where a metal centre resides in both DNA grooves. Such systems exhibit increased affinity and enhanced binding specificity towards particular DNA structures.[^20^, ^21^]

These threading intercalators have been extensively studied in optical tweezer experiments in which a single molecule of DNA tethered between two optically trapped beads provides a method to directly measure the position and force on the beads. Using this technology, the very slow DNA threading kinetics of the complexes were accelerated so that they can be monitored in real time.^[22]^ This facilitated the quantification of binding and dissociation kinetics, while simultaneously probing any concomitant DNA structural changes. These experiments revealed a two‐step “thread‐and‐lock” mechanism, whereby threading results in a stable “locked” complex that is exceptionally slow to dissociate. They also disclosed that ancillary ligands play a large role on this thread‐and‐lock mechanism; complexes incorporating phen ancillary ligands show locked binding, whilst analogues containing more compact bpy ancillary ligands require no DNA extension to de‐thread.^[23]^ Again, chirality has a profound role in the binding affinity of threading events.^[24]^

Although their threading interaction makes these complexes attractive candidates as optical probes for DNA, their use in such applications is restricted by the fact that they are not intrinsically cell permeant. By contrast, in related work, the Thomas group has demonstrated that certain dinuclear ruthenium complexes of the form [{Ru(L)_2_}_2_‐μ‐tpphz]^4+^ (where L is a dipyridyl related ancillary ligand and tpphz=tetrapyrido[3,2‐a:2′,3′‐c:3′′,2′′‐ h:2′′′,3′′′‐j]phenazine) *are* taken up by live cells.^[25]^ Several derivatives have been developed as DNA probes for live cell super‐resolution microscopy, anticancer therapeutics, and antimicrobial theranostics.[^26^, ^27^, ^28^, ^29^] Consequent structural studies on [{(Ru(bpy)_2_}_2_‐μ‐(tpphz)]^4+^ (bpy=2,2′‐bipyridine) and related structures (**1** ^4+^, Figure 1), have demonstrated they possess structural specificity for the DNA quadruplex structure such as the human telomeric repeat sequence and chirality dependant loop threading into quadraplexes.^[30]^ Whilst spontaneous intercalation of **1** ^4+^ into stable duplexes is not observed,^[31]^ more recent NMR‐based studies revealed that it can bind to a short B‐DNA oligonucleotide through threading. Furthermore, due to the short and rigid tpphz bridging ligand, insertion of this “minimal threader” is acutely dependent on metal centre chirality. Whilst Λ,Λ‐1^4+^ displays locked threading, duplex‐bound Δ,Δ‐1^4+^ equilibrates between a groove‐bound and threaded state.^[32]^

These observations suggest that threading of Λ,Λ‐**1** ^4+^ could occur with longer destabilized duplex sequences. To investigate this possibility, we combine optical tweezers with laser scanning confocal microscopy (LSCM) to directly investigate the kinetics and binding modes of the three stereoisomers of **1** ^4+^, while simultaneously probing their luminescent properties. Through these experiments we found that Λ,Λ‐**1** ^4+^ functions as a “lockable” luminescent stain for duplex DNA.

## Results and Discussion

### DNA Binding Occurs in Two Phases

**Figure 2:**
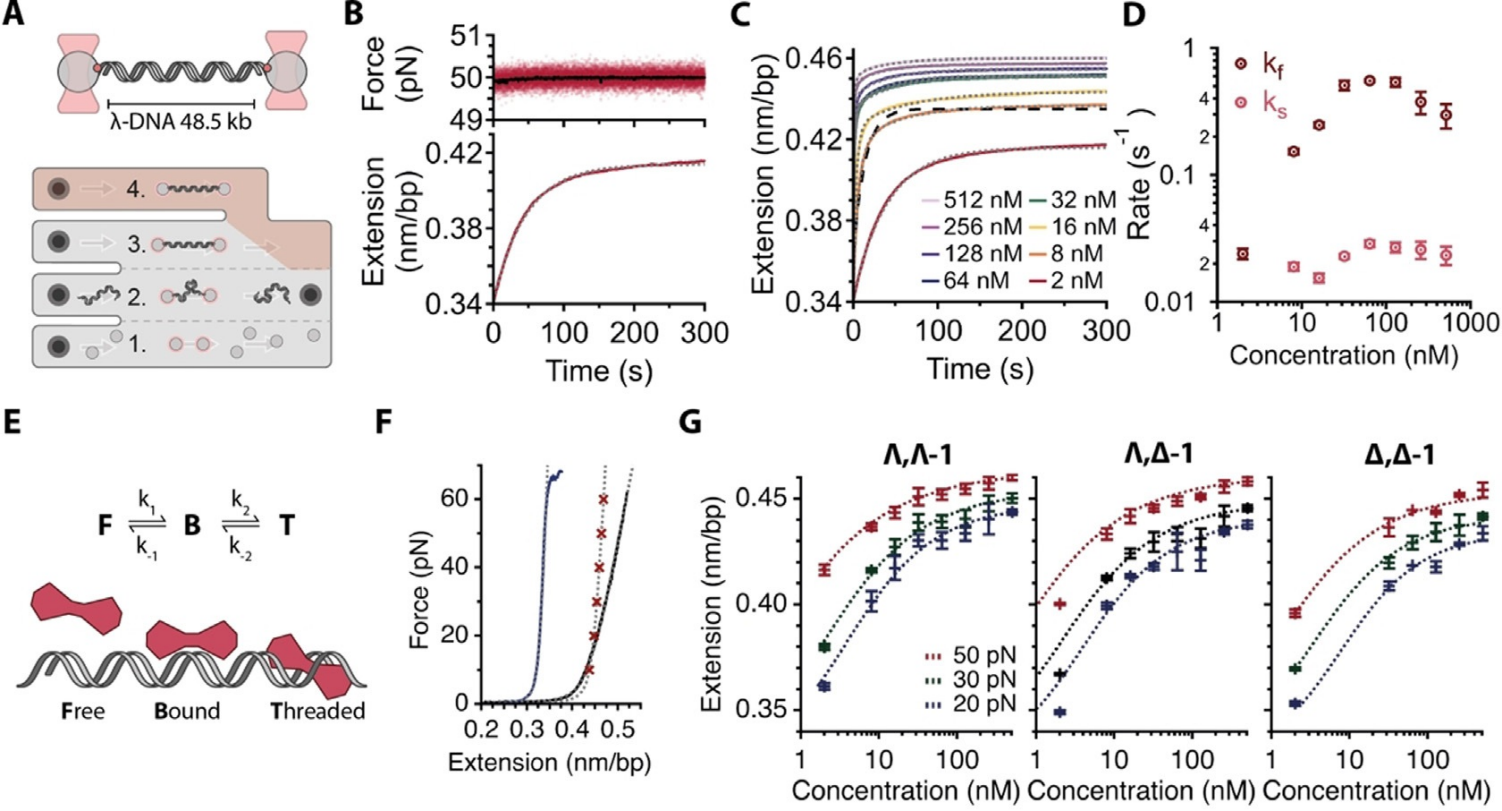
DNA intercalation occurs via a two‐step mechanism. A) Optical tweezers experiment set‐up (top) and microfluidic flow cell (bottom). λ‐DNA is tethered between trapped beads (**1**., **2**.) and held at constant force in the presence of intercalator (**4**.). To measure dissociation, DNA molecule is moved to position in flow cell with buffer only, 20 mM Tris‐HCl pH 7, 100 mM NaCl (**3**.). B) Typical extension‐time experiment with 8 nM Λ,Λ‐**1** ^4+^. DNA is held at a constant 50 pN via a distance feedback loop (top, red points 60 Hz raw data, black line 100 point smoothed). DNA intercalation causes an increase in extension over time (bottom). Data fit with a single exponential time dependence (light grey dashed line). C) Representative extension‐time traces for Λ,Λ‐**1** ^4+^ intercalation at 50 pN at different concentrations with corresponding fits (light grey dashed lines). At concentrations of 8 nM and higher data was fit to double exponential time dependence. A single exponential fit at 8 nM is also displayed for comparison (dark dashed line). D) Observed rates for Λ,Λ‐**1** ^4+^ at 50 pN, obtained from fits to extension‐time traces, as a function of concentration. *k*_f_ displays clear concentration dependence whereas *k*_s_ is concentration independent. E) Schematic of proposed model for two step intercalation (F) Equilibrium DNA extension measured in the absence of intercalator (blue line), at saturating SYTOX Orange concentration (1 μM, grey line) and at saturating concentration of Λ,Λ‐**1** ^4+^ (1 μM, red crosses), both fit with extendable worm‐like chain model (grey dashed). G) Equilibrium extensions as a function of intercalator concentrations from 2 nM to 512 nM at 20, 30 and 50 pN for the three stereoisomers, each fit with the MVH binding isotherm (dashed lines).

The interaction of the chloride salt of Λ,Λ‐**1^4+^** with a single molecule of λ‐DNA was first investigated. A DNA dumbbell was assembled using a microfluidic laminar flow cell (Figure 2 A, channels 1–3), and held at a constant force (20–50 pN) in the presence of the complex (2–512 nM) (Figure 2 A, channel 4). At these forces, the DNA remains double‐stranded, however the stability is reduced at higher forces (>60 pN). The kinetics of the interaction could be readily monitored through the increase in DNA extension as a function of time (Figure 2 B and Supplementary Data Figure 1) as probe binding increases the spacing between base pairs.

At the lowest concentration (2 nM), the resulting time traces were fit to a single exponential increase [Figure 2 C, Eq. (1)], whereas at all higher concentrations (≥8 nM) double exponentials were required [Figure 2 C, Eq. (2)]. The fits show that the rate constant for the fast phase, *k*_f_, increases with concentration of Λ,Λ‐1^4+^, whereas the slow phase rate constant, *k*_s_, remains approximately constant (Figure 2 D, Supplementary Data Figure 2).

These experiments were then repeated with the two other stereoisomers. As with Λ,Λ‐**1** ^4+^, at low concentration (2 nM) the extension time traces for Λ,Δ‐**1** ^4+^ and Δ,Δ‐**1** ^4+^ were fit with a single exponential [Supplementary Data Figure 1, Eq. (1)], whereas at higher concentrations (≥8 nM) double exponentials were required [Supplementary Data Figure 1, Eq. (1)]. Again, the fits for Λ,Δ‐**1** ^4+^ and Δ,Δ‐**1** ^4+^ show that the fast phase rate constant increases with concentration of **1** ^4+^, whereas the slow phase rate constant is concentration independent.

These observations indicate that all three stereoisomers interact with DNA in a multi‐step process, consistent with a minimal two‐step kinetic model (Figure 2 E) involving a rapid, concentration‐dependent binding phase and a slow, concentration independent unimolecular phase.

Previously published single molecule experiments on threading mono‐intercalated Ru^II^ complexes, such as [{(Ru(LL)_2_}_2_(bidppz)]^4+^, show that they bind to DNA through a single state mechanism.^[22]^ The exception is a dinuclear complex containing a more flexible central bridging ligand, which initially binds to duplex through a conventional intercalated state before threading occurs.^[33]^ On the other hand, bulk optical titrations and linear dichroism experiments on the original, more rigid, threader are consistent with a two‐state mechanism, in which groove binding proceeds threading.[^24^, ^34^, ^35^] Given these observations, and the facts that; **1** ^4+^ contains a shorter inflexible tpphz bridging ligand, binds extended stable duplex sequences through groove binding, and binds unstable duplexes through both groove binding and threading, we attribute the rapid binding phase observed in these experiments to groove binding and the slow phase to threading (Figure 2 E).

### Ru‐bpy Stereoisomer Intercalators Differentially Affect DNA Elasticity

Next, we determined the equilibrium DNA extension at a saturating concentration of **1** ^4+^ at various forces (10–60 pN, Figure 2 F), and fit those to the extensible wormlike‐chain model [Eq. (7)] to obtain the contour length, persistence length and stretch modulus of fully intercalated DNA for each stereoisomer (Figure 2 F, Table 1). All the stereoisomers induce a similarly pronounced increase in duplex contour length, which is expected from an intercalative interaction, comparable to the widely‐used SYTOX Orange (Table 1, Figure 2 F). However, whilst Λ,Δ‐**1** ^4+^ and Δ,Δ‐**1** ^4+^ produce significant decreases in persistence length and stretch modulus, addition of Λ,Λ‐**1** ^4+^ results in only slight changes in these parameters compared to duplex DNA (Table 1). The substantial increase in DNA elasticity produced by addition of Λ,Δ‐**1** ^4+^ and Δ,Δ‐**1** ^4+^ is consistent with our NMR model which shows that each bound Δ Ru^II^ centre has a destabilizing effect on DNA structure.^[32]^ Interestingly, Λ,Λ‐**1** ^4+^ preserves the DNA structural properties far better than SYTOX Orange (Table 1 and Figure 2 F).

**Table 1:** eWLC DNA parameters at saturating intercalator concentrations.

	Contour Length [μm]	Base Pair Rise [Å/bp]	Persistence Length [nm]	Stretch Modulus [pN nm^−1^]
dsDNA	16.19±0.05	3.3±0.1	46±1	1360±30
Λ,Λ‐**1** ^4+^	22.12±0.05	4.6±0.1	42±3	1310±40
Λ,Δ‐**1** ^4+^	21.83±0.10	4.5±0.1	31±4	960±41
Δ,Δ‐**1** ^4+^	21.48±0.15	4.4±0.1	24±1	930±20
O‐SYTOX	21.93±0.24	4.5±0.1	18±1	340± 4

### Force Stretching DNA Increases Ru‐bpy Intercalation Affinity

To determine the force dependence of the intercalator binding affinity, we measured the equilibrium extension (*L*_eq_) of each stereoisomer at increasing intercalator concentrations and three constant forces (20, 30 and 50 pN, Figure 2 G). The resulting curves were fit to the well‐established McGhee‐von Hippel, MVH, binding isotherm [Eq. (8)],[^36^, ^37^] which allows estimation of the binding site size per molecule (*n*) and force dependent *K*_d_ (Table 2). The fits show that all three stereoisomers bind DNA extremely tightly (low nanomolar *K*_d_), and that force stretching further increases affinity (lower *K*_d_). The binding site size, *n*, of all three stereoisomers increases with increasing force. For both the Λ,Λ‐**1^4+^** and Λ,Δ‐**1^4+^** stereoisomers, the binding site size increases from ≈1.5 bp (20 pN) to ≈2 bp (50 pN), suggesting that threading is increasingly likely at high forces with a very high degree of threading occurring at high force. The binding site size of Δ,Δ‐**1^4+^** is consistently smaller, increasing from ≈1.3 bp (20 pN) to only ≈1.8 (50 pN).This is consistent with our previous NMR studies showing that duplex‐bound Δ,Δ‐**1^4+^** is equilibrating between groove binding and threaded states.^[32]^

**Table 2:** Calculated K_d_ and binding site size, n, at different forces. Parameters calculated from MVH binding analysis for 20 pN, 30 pN and 50 pN forces. Error corresponds to one standard deviation of fitting.

	Force [pN]	*K*_d_ [nM]	*n*
Λ,Λ‐**1** ^4+^	20	5.6±0.3	1.5±0.1
	30	3.7±0.2	1.7±0.1
	50	1.3±0.1	2.2±0.1
Λ,Δ‐**1** ^4+^	20	5.6±0.4	1.5±0.1
	30	3.6±0.3	1.7±0.1
	50	1.4±0.1	2.0±0.1
Δ,Δ‐**1** ^4+^	20	9.6±0.1	1.3±0.2
	30	5.0±0.1	1.5±0.1
	50	2.6±0.1	1.8±0.1

### Parametrisation of the Minimal Two‐Step Binding and Threading Kinetic Model

**Figure 3:**
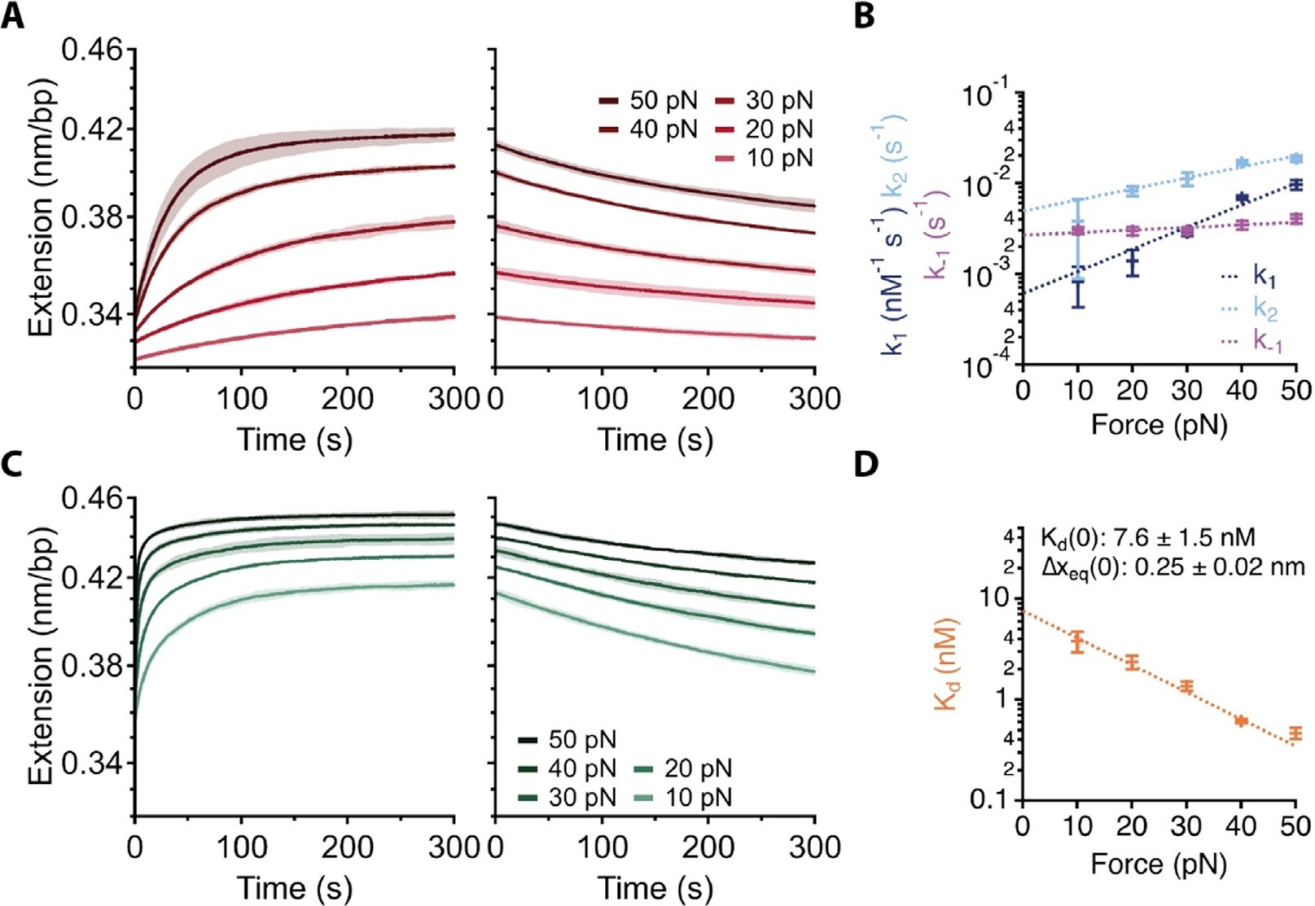
Determining elementary rates. A) Average Λ,Λ‐**1^4+^** extension‐time traces at 2 nM of association (left) and dissociation (right) for forces 10–50 pN. (*n*=3, dark line=mean, light shaded=two standard deviations) (B) Force dependant elementary rate constants, *k*_1_ and *k*_2_, and observed dissociation rate constant, *k*_−1_. Fit with exponential force dependence [Eq. (4)] and extrapolated to give zero force values. (Error bars=s.d.) (C) Average Λ,Λ‐**1** ^4+^ extension‐time traces at 32 nM of association (left) and dissociation (right) for forces 10–50 pN. (*n*=3, dark line=mean, light shaded=two standard deviations) (D) Force dependant calculated equilibrium constants, fit with exponential force dependence [Eq. (4)] and extrapolated to give zero force values.

To determine the parameters of the minimal kinetic model (*k*_1_, *k*_−1_ and *k*_2_, Figure 2 D), we took advantage of the fact that at low concentration (2 nM) initial binding is rate limiting (Figures 2 B and C), enabling us to determine the corresponding rate constants (*k*_1_ and *k*_−1_) under these conditions for all three stereoisomers. First, the DNA extension was measured for 5 minutes at forces ranging 10–50 pN (Figure 3 A, left and SI Figure 3 A). A single exponential fit yields the observed binding rate constant *k*_f_ [Eq. (4)].

The same DNA molecule was then moved to a buffer‐only channel (Figure 2 A, Position 3) to measure the dissociation‐induced DNA shortening for 5 minutes (Figure 3 A, right and SI Figure 3). A single exponential fit directly yields the dissociation rate constant *k*_−1_ (Figure 2 D). The binding rate constant *k*_1_ is then calculated as indicated by Equation (4). We then determined the binding and dissociation rate constants as a function of force (10–50 pN). The data show that increasing force results in faster binding (Figure 3 B, *k*_1_), whereas dissociation is largely force independent (Figure 3 B, *k*_−1_). Similar results were obtained for all stereoisomers (Supplementary Data Figure 3), suggesting that increasing the separation between base pairs at higher forces facilitates binding but not dissociation. A fit to the Bell‐Evans equation [Eq. (5)] yields the zero force rate constants and the location of the transition state barrier (Figure 3 B and Table 3). The fits show that to reach the binding transition state the DNA must be stretched by Δ*x*_1_≈0.2–0.3 nm (Table 3). Conversely, reaching the dissociation transition state requires no further deformation of the DNA (Table 3, Δ*x*_−1_≈0 nm). The binding‐induced DNA elongation provoked by **1** ^4+^ is comparable to the data^[23]^ reported for Δ,Δ‐[{(Ru(bpy)_2_}_2_(bidppz)]^4+^ showing that the structural perturbation required to thread a [Ru^II^(bpy)_2_] moiety is similar in both systems, as might be expected. Interestingly all three stereoisomers have a Δ*x*_−1_ of ≈0. To determine the rate constant for the second binding mode (*k*_2_), we performed measurements at high concentration (32 nM) for all stereoisomers (Figure 3 C and Supplementary Data Figure 4). A double exponential fit of the binding curves yields the two observed association rate constants, *k*_f_ and *k*_s_. At high concentrations, the fast phase becomes too fast to be fit accurately, therefore we fixed *k*_f_ to the *k*_1_ values obtained previously in the low concentration experiments adjusted to the higher concentration, which enables us to accurately fit for *k*_s_ (Figure 3 C and Supplementary Data Figure 4 and Supplementary Data Table 1–2). Interestingly, even at high complex concentration (32 nM) dissociation remains single exponential, with fits yielding the same observed dissociation rate constant (*k*_off_), consistent with the low concentration experiments (Figure 3 C and Supplementary Data Figure 4). Furthermore, the dissociation rates for all stereoisomers are comparable at low and high concentrations (*k*_−1_≈*k*_off_) (Supplementary Data Figure 3 and Supplementary Data Figure 4), suggesting that dissociation from the threaded state is very slow [*k*_−2_≈0, Eq. (4)].

**Table 3:** Zero force elementary rate constants and equilibrium parameters for different stereoisomers. Parameters calculated from kinetic measurements. Error corresponds to one standard deviation.

	*k*_1_(0) [×10^5^ M^−1^ s^−1^]	Δ*x*_1_ [nm]	*k*_−1_(0) [×10^−3^ s^−1^]	Δ*x*_−1_ [nm]	*k*_2_(0) [×10^−3^ s^−1^]	Δ*x*_2_ [nm]	*K*_d_^0^ [nM]	Δ*x*_eq_ [nm]	*n*
Λ,Λ‐1	6.1±2.4	0.23±0.01	2.6±0.3	0.05±0.01	4.9±0.8	0.12±0.02	7.6±1.5	0.25±0.02	2.1±0.2
Λ,Δ‐1	10.5±1.2	0.22±0.01	9.4±0.4	−0.02±0.01	10.3±1.5	0.07±0.02	10.2±1.0	0.25±0.01	2.2±0.2
Δ,Δ‐1	11.6±1.5	0.20±0.01	5.3±0.3	0.05±0.01	5.6±0.6	0.08±0.01	8.9±0.9	0.24±0.01	2.2±0.2

The resulting fits show that increasing the force facilitates both groove binding, as indicated by the increasing *k*_1_, and also threading, indicated by the increasing *k*_2_. Comparison of the calculated values of Δ*x*_1_ and Δ*x*_2_ (Table 3) suggests that the DNA is deformed by ≈2 Å in the initial groove binding step and an additional ≈1 Å during the slow threading step. As such, both steps are aided by increased extension of the DNA. Our NMR‐based studies,^[32]^ and that of others,^[38]^ have shown that groove binding of this class of compound leads to steric clashes within the minor groove. Thus, we attribute the cause of the groove‐binding induced extensions to these interactions. At low concentration (2 nM) the initial groove binding is rate limiting however at higher concentrations (≥8 nM) the second step becomes rate limiting with groove binding occurring faster than threading (Figure 3 B).

Based on the dissociation and association rate constants, we calculate the equilibrium constant of the initial binding phase as a function of force (Figure 3 D, Supplementary Data Figure 3 and Table 3). As expected, the dissociation constant decreases with increased force, indicating tighter binding. The calculated position of the transition state barrier (Δ*x*_eq_) confirms a ≈2.5 Å distortion for binding. From this and the previously determined contour lengths at saturation, *L*_eq_, the site size per bound ligand, *n*, can be estimated for each stereoisomer as ≈2 base pairs [Eq. (6)], in agreement with the MVH analysis (Table 2). Significantly, all three complexes display exceptional high binding affinities (≤10 nM) at the extrapolated zero‐force values. Notably, the estimated *K*_d_ for complex Λ,Λ‐**1** ^4+^ is six‐fold smaller than that reported for Δ,Δ‐[{(Ru(phen)_2_}_2_(bidppz)]^4+^ and is—as far as we are aware—the lowest reported for *any* mono‐intercalator. This enhanced binding affinity of Λ,Λ‐**1** ^4+^ compared to [{(Ru(LL)_2_}_2_(bidppz)]^4+^ complexes can be attributed to differences in the dissociation rates of the systems. As discussed above, the DNA association rates for all the stereoisomers of **1** ^4+^ are close to, or faster, than those of [{(Ru(bpy)_2_}_2_(bidppz)]^4+^, but the dissociation rate of Λ,Λ‐**1** ^4+^ is closer to that of [{(Ru(phen)_2_}_2_(bidppz)]^4+^. We hypothesize that the lower DNA distortion required to thread a [Ru^II^(bpy)_2_] moiety along with a close match between the width and steric demands of duplex DNA and the bound minimal threader Λ,Λ‐**1** ^4+^ leads to optimization of association and dissociation rates. However, further related compounds would need to be validated to confirm this.

### Luminescent Imaging of Intercalated DNA

**Figure 4:**
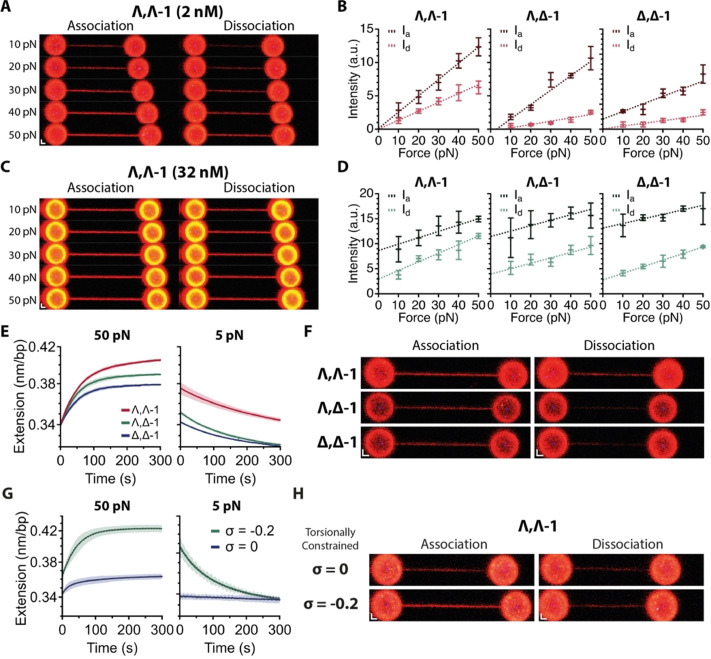
Comparison of the imaging properties of the different stereoisomers. A) Confocal images of Λ,Λ‐**1** ^4+^ intercalator bound DNA held between the two trapped beads. The DNA was imaged after incubation with 2 nM Λ,Λ‐**1** ^4+^ under a constant force clamp (10–50 pN) for 5 minutes (“Association”, left). The same piece of DNA was then imaged after a further 5 minutes held at constant force (10–50 pN) in the buffer only channel (“Dissociation”) Scale bar=1 μm (B) Quantification of emission intensities of labelled DNA after association in the presence of 2 nM intercalator (I_a_, dark red) and dissociation (I_d_, light red) with DNA held at each force, for each stereoisomer. () Confocal images of Λ,Λ‐**1^4+^** intercalator bound DNA held between the two trapped beads. The DNA was imaged after incubation with 32 nM Λ,Λ‐**1** ^4+^ under a constant force clamp (10–50 pN) for 5 minutes (“Association”, left). The same piece of DNA was then imaged after a further 5 minutes held at constant force (10–50 pN) in the buffer only channel (“Dissociation”) Scale bar=1 μm (***D***) Quantification of the fluorescent intensities of the DNA after association in the presence of 32 nM intercalator (I_a_, dark green) and dissociation (I_d_, light green) with DNA held at each force, for each stereoisomer. E) Average extension‐time trajectories (*n*=3) comparing the three stereoisomers for the “bind and lock” protocol. First the DNA is clamped at 50 pN for 5 minutes in the presence of 1 nM of **1** ^4+^, (left). The DNA is then moved to buffer only channel and held at 5 pN for 5 minutes (right). F) Confocal images comparing the emission of each DNA‐bound stereoisomer during the “bind and lock” protocol. A confocal scan is taken after a 5 minute 50 pN clamp with 1 nM intercalator (“Association”, left) and another scan after a 5 minute 5 pN clamp in the buffer only channel (“Dissociation”, right) Scale bar=1 μm (G) Average extension‐time trajectories (*n*=3) for binding and dissociation of Λ,Λ‐**1** ^4+^ to torsionally constrained but not supercoiled DNA (*σ*=0) and negatively supercoiled DNA (*σ*=−0.2). H) Corresponding confocal images from (G), following association and dissociation of Λ,Λ‐**1** ^4+^ in “bind and lock” protocol.

Despite their light‐switching properties upon DNA binding, the distinct emission properties of the different isomers of **1** ^4+^ have never been investigated directly. Thus, using LSCM, we imaged the DNA in the presence of 2 nM of each stereoisomer of **1** ^4+^ from 10–50 pN (Figure 3 A, Supplementary Data Figure 2). After 5‐minute incubation, a single confocal scan was taken (Figure 4 A, left, Supplementary Data Figure 5). Next, the same molecule of DNA was moved to the buffer only channel (Figure 2 A, Position 3.) and held at constant force for 5 minutes before taking a second confocal scan (Figure 4 A, right, Supplementary Figure 5). Relative DNA emission intensity per base pair was calculated after the association step, **I_a_**, and after the dissociation step, **I_d_**, and plotted against force for each stereoisomer (Figure 4 B). A clear force dependent increase in DNA luminescence is observed, consistent with the force dependent increase in equilibrium extension. As expected, the emission intensity is greatest after the association step, and reduced after the 5‐minute dissociation step. Although this is a general trend, there are some very clear differences between stereoisomers. Λ,Λ‐**1^4+^** displays the greatest emission intensity at all forces after the initial association step and most interestingly retains its higher intensity after the dissociation step (Figure 4 A, B). This suggests a larger population of permanently‐bound, threaded Λ,Λ‐**1^4^**.

We next performed the same experiments at much higher intercalator concentration (32 nM). As before the DNA luminescence displayed a clear force dependence; however, even at 10 pN a high level of emission was observed (Figure 4 C, D). Furthermore, all three isomers displayed comparable DNA emission intensities despite the fact that Λ,Λ‐**1^4+^** displays a higher equilibrium extension than Λ,Δ‐**1^4+^** and Δ,Δ‐**1^4+^**. Although we cannot directly distinguish between emission from the groove bound versus threaded state this suggests that a significant amount of emission is coming from non‐intercalating molecules, which is consistent with previous reports showing that the groove bound complex is also emissive.^[31]^ Again, after a 5‐minute dissociation step, Λ,Λ‐**1** ^4+^ displays the highest level of emission. This provides further evidence for a population of luminescent molecules bound through a non‐intercalating mode which rapidly dissociate to leave the stable threaded population.

Based on the observed force dependence of both association and dissociation, we compared the three stereoisomers in a “load and lock” assay of DNA labelling. First, we incubated the DNA with a low concentration of intercalator, 1 nM, clamped at high force, 50 pN, to maximize intercalative binding (Figure 4 E, left) and after 5 minutes we performed a confocal scan (Figure 4 F, left) to confirm “loading” of the complex onto DNA before moving to the buffer only channel and clamping the DNA at low force (10 pN) (Figure 4 E, right). At this low force we reasoned that the rate of dissociation of any threaded **1** ^4+^ would be negligible compared to its groove bound form. Consequently, after 5 minutes in the buffer only channel, we took another confocal scan (Figure 4 F, right). Consistent with our assumptions, and the threading capabilities of the stereoisomers, we found that, after this protocol, little emission remained in samples of DNA exposed to Λ,Δ‐**1^4+^** and Δ,Δ‐**1^4+^**, yet DNA loaded with Λ,Λ‐**1^4+^** still remained brightly luminescent, indicating that a large proportion of this stereoisomer remains “locked” onto the duplex through threading.

This is in sharp contrast to the extremely rapid dissociation of SYTOX observed following the same protocol (Supplementary Data Figure 6).

To investigate whether ionic strength regulates probe binding, we measured the association and dissociation rate constants at low and high NaCl concentrations (Supplementary Data Figure 7 A). The data show that at 50 pN, higher ionic strengths (200 and 500 mM NaCl) slow probe binding, as evidenced by a ≈3‐fold reduced association rate constant (*k*_a_), whereas the dissociation rate constant (*k*_d_) remains unchanged (Supplementary Data Figure 7 B). These results are consistent with a role of metal ions in shielding the DNA negative charge, thereby reducing the affinity of the positively charged ruthenium compounds. Consequently, we also observe a corresponding decrease in DNA extension after probe‐binding (Supplementary Data Figure 7 C).

To further demonstrate the potential application of these compounds, we measured the kinetics of Λ,Λ‐**1** ^4+^ binding to torsionally constrained DNA (Figure 4 G,H). Binding is dramatically impaired in the presence of torsionally constrained DNA (*σ*=0, Figure 4 G,H). Interestingly, when the DNA is negatively supercoiled, DNA binding is rescued (*σ*=−0.2, Figure 4 G,H). The data from binding and dissociation kinetic experiments show that Λ,Λ‐**1** ^4+^ preferential threads in negatively supercoiled DNA compared to relaxed torsionally constrained DNA (Figure 4 G,H).

## Conclusion

This work presents the highest affinity mono‐intercalating DNA binding complex to date, and represents the shortest linked Ru^II^(dppz) complex capable of DNA threading. Given the length of tpphz and the diameter of duplex DNA, any further reduction in the threading linker length could not be accommodated across the DNA helix (Figure 1).

The observation that all three stereoisomers of **1** ^4+^ bind duplex DNA in two steps is consistent with previous studies^[33]^ initial groove bound or unthreaded intercalated states prior to irreversible threading (*k*_−2_≈0). Similar to the Michaelis–Menten mechanism, our calculations of *K*_d_ are only valid under the assumption of rapid pre‐equilibrium, which is supported by our measurements of *k*_f_ and *k*_s_ (Figure 2 D). Due to the rigid linker structure of **1** ^4+^, we can rule out the possibility of unthreaded intercalation and attribute the fast step to groove binding; the fact that this step is concentration and force dependent is intriguing. Previous studies on the interaction of [Ru^II^ (LL)_2_(dppz)]^2+^ and derivatives with DNA have frequently shown cooperative binding effects, often driven by stacking interactions of ancillary ligands. Thus, it seems likely that at higher complex loading ratios, charge neutralization effects involving the electronegative minor groove facilitate similar interactions between individual groove‐bound **1** ^4+^ cations. The strong force dependence of the fast initial binding step and addition force dependence of the slower threading step suggests that majority of DNA deformation occurs during the groove binding step, with a smaller additional deformation required for threading.

The studies herein highlight the influence of chirality on threading interactions as it is clear that individual stereoisomers exhibit different binding behaviors into stretched DNA. Due to unfavourable steric interactions, incorporation of each Δ metal centre into the threader causes increased DNA flexibility, seen as a reduction in both DNA persistence length and stretch modulus of bound DNA, and also a reduction in binding affinity (Table 1). This means that, of the three stereoisomers, Λ,Λ‐**1** ^4+^ displays the optimized thermodynamic and kinetic threading stability, binding to DNA with highest reported affinity for a mono‐intercalator. As a consequence, once Λ,Λ‐**1** ^4+^ is loaded on to stretched DNA, on removal of the stretching force it is effectively irreversibly threaded into duplex (Figure 4 E).

As a result of this load and lock approach, single DNA molecules can be permanently labelled by exposure to a low concentration of luminescent small‐molecule dye without the demanding, and often structurally disruptive, protocols required to covalently attach small molecules or large biomacromolecule structures such as fluorescent proteins. As this dye only becomes brightly luminescent on binding to DNA, and its groove bound form is easily washed away, the protocol we describe facilitates high‐contrast single molecule imaging of duplex DNA through optical microscopy. As this class of dye is compatible with super‐resolution SIM and STED techniques, a range of possible imaging applications is apparent, such as the intriguing possibility of developing a probe to specifically visualize destabilized, transcriptionally active, DNA. This possibility is further supported by the observation of the dramatic reduction in binding to torsionally constrained DNA and the rescue in binding to negatively supercoiled DNA. These studies will also inform the structural optimization of their cell permeant analogues to facilitate the development of new and novel imaging probes and therapeutic leads. Work addressing these issues will form the basis of future reports.

## Conflict of interest

The authors declare no conflict of interest.

## Supporting information

As a service to our authors and readers, this journal provides supporting information supplied by the authors. Such materials are peer reviewed and may be re‐organized for online delivery, but are not copy‐edited or typeset. Technical support issues arising from supporting information (other than missing files) should be addressed to the authors.

Supporting Information
