# Supplementary material for: A Minimal Load‐and‐Lock RuII Luminescent DNA Probe
Source: Angew Chem Int Ed Engl. 2021 Aug 11;60(38):20952–9. doi: 10.1002/anie.202108077 (PMC8518596; doi:10.1002/anie.202108077)
Supplement: Supplementary file 1 — Supporting Information [file ANIE-60-20952-s001.pdf]

Angewandte Chemie
Eine Zeitschrift der Gesellschaft Deutscher Chemiker

Supporting Information

A Minimal Load-and-Lock Ru^{II} Luminescent DNA Probe

Matthew D. Newton[+], Simon D. Fairbanks[+], Jim A. Thomas, and David S. Rueda**

anie_202108077_sm_miscellaneous_information.pdf

SUPPORTING INFORMATION

Content:

Materials and Methods:	pp. 2-5
Supplementary Figures:	pp. 6-14
Supplementary Tables:	p. 15
Supplementary References:	p. 16

Materials and Methods

Synthesis and isomeric separation of compounds

$[\{Ru(bpy)_2\}_2(tpphz)](PF_6)_4$ was synthesized, converted to its chloride salt using a Dowex anion-exchange resin, and resolved into its three stereoisomers using literature methods.

The chiral purity of the compounds was verified by circular dichroism (**Supplementary Data Figure 8**). Compounds were further characterized by 2D-NMR (**Supplementary Data Figure 9**). Concentrations were determined by spectrophotometry using previously determined absorption coefficients for the chloride salt in water.

Preparation of DNA substrates

λ-DNA was used to tether between the trapped beads due to its extensive characterization and use in optical tweezers experiments to study DNA properties, small molecule binding and DNA-protein interactions. For experiments with torsionally unconstrained DNA, λ-DNA was purchase from Life Technologies Limited (SD0011) and the ends biotinylated through addition of biotinylated nucleotides (B-dCTP LifeTechnologies Limited #19518018, B-dATP Life Technologies Limited #9524016) by Klenow polymerase (Klenow Fragment 3' to 5' exo- NEB M0212M) was used to fill in the overhanging *cos* sites as previously described[1–3].

For experiments with torsionally constrained and negatively supercoiled DNA, λ-DNA was capped by ligation of a DNA hairpin end cap containing multiple biotin modified T bases (**Supplementary Data Table 3**) as previously described[4,5].

Optical tweezers with microfluidics and fluorescent microscopy

All experiments were performed on the commercially available Lumicks C-trap with dual optical traps, microfluidic flow cell and integrated confocal microscopy. The flow cell was cleaned with bleach and sodium thiosulphate before being flushed with buffer and passivated with Pluronic F128 (0.5\% w/v in reaction buffer) flowed over 30 min period. For each intercalator concentration used the tubing and channel of the flow cell (Figure 2A, 4.) were passivated by flowing > 2 ml of solution over 30

minutes. Biotinylated λ-DNA was tethered between two optically trapped 4.42 μm streptavidin coated polystyrene beads (0.5% w/v, Spherotech, Cat# SVP-40-5).

For experiments with torsionally unconstrained DNA, the presence of a single molecule of tethered DNA was verified by performing a force-distance curve from ~5-17 μm and observing the expected contour length (~16 μm) and overstretching plateau at ~60 pN. For experiments with torsionally constrained DNA, the presence of a single molecule of un-nicked torsionally constrained DNA was verified by performing a force-distance curve from ~5 - 17 μm and observing the expected contour length (~16 μm) and higher overstretching plateau at ~120 pN compared to torsionally unconstrained DNA. To generate negatively supercoiled DNA torsionally constrained molecules were repeatedly held at ~ 150 pN for a few seconds before returning to low force. The generation of negative supercoiling was observed by an increase in the extension at forces > 40 pN and the extension at 70 pN was used to determine the degree of supercoiling based on previously described calibration[4].

For confocal imaging we used an excitation wavelength of 488 nm with a 0.1 ms pixel dwell time, 100 nm pixel size. The emission was detected with a red 640 LP filter and APD single photon counter.

Unless otherwise stated experiments were performed in buffer containing 20 mM Tris-HCl pH 7, 100 mM NaCl.

Kinetic Analysis

At low concentration (2 nM) extension-time data was fit to a single exponential time dependence whereas at higher concentrations a double exponential was required.

$$L(t) = L_0 + L_{eq}\left(1 - e^{-k_f t}\right) \quad \textbf{(Eq. 1)}$$

$$L(t) = L_0 + L_f\left(1 - e^{-k_f t}\right) + L_s(1 - e^{-k_s t}) \quad \textbf{(Eq. 2)}$$

For dissociation experiments all data was fit to a single exponential decay.

$$L(t) = L_0 - L_{eq}(1 - e^{-kt}) \quad \textbf{(Eq. 3)}$$

Intercalation occurs in a two-step mechanism defined by four elementary rates (**Figure 2D**). Based on this mechanism the observed rates (k_f and k_S) can be used to determine the elementary rates by fitting to the following relationships:

$$k_f = k_1 C + k_{-1},\; k_s = \left(\frac{k_1 C}{k_1 C + k_{-1}}\right) k_2 + k_{-2} \qquad \textbf{(Eq. 4)}$$

The zero force elementary rates, k_0, and corresponding lengths of DNA deformation, *x*, are obtained by fitting of the force dependant rates as previously described[25].

.
$$k(F) = k_0 e^{-\frac{Fx}{k_b T}} \qquad \textbf{(Eq. 5)}$$

An estimate of the site size bound per ligand can be calculated from the per base extension at equilibrium, Δx, and the per base increase in contour length at saturation:

$$n = \frac{\Delta x}{\Delta L_{eq}} \qquad \textbf{(Eq. 6)}$$

Extension analysis

The dsDNA and saturating intercalator DNA extension (nm/bp) as function of force (pN) were fit with the extensible Worm-like Chain model.

$$d(F) = L_0 \times \left(1 - \frac{1}{2}\sqrt{\left(\frac{k_B \times T}{L_p \times F}\right)} + \frac{F}{K_0}\right) \qquad \textbf{(Eq. 7)}$$

Where L_0 = contour length (nm/bp), L_p = persistence length (nm), K_0 = elastic modulus (pN), k_B = boltzman constant and T = temperature (K)

The final equilibrium extension as a function of concentration and force, $L_{eq}(F,C)$, was fit to the McGhee-von Hippel binding isotherm as previously described[25].

$$L_{eq}(F,C) = L_0(F) + \Theta(F,C) \times \delta L_{sat}(F),\; \Theta(F,C) = \frac{C}{K_d(F)} \times \frac{n \times (1-\Theta)^n}{(1-\Theta+\frac{\Theta}{n})^{n-1}} \qquad \textbf{(Eq. 8)}$$

Intensity Analysis

To quantify the DNA fluorescence intensity the average pixel intensities across 20 columns was calculated and the resulting intensity profile fit to a gaussian

distribution. The area under the gaussian was divided by the pixel size to give DNA intensity per micron. This was then multiplied by the DNA length in microns to give a measure of the intensity per bp.

Supplementary Data Figures

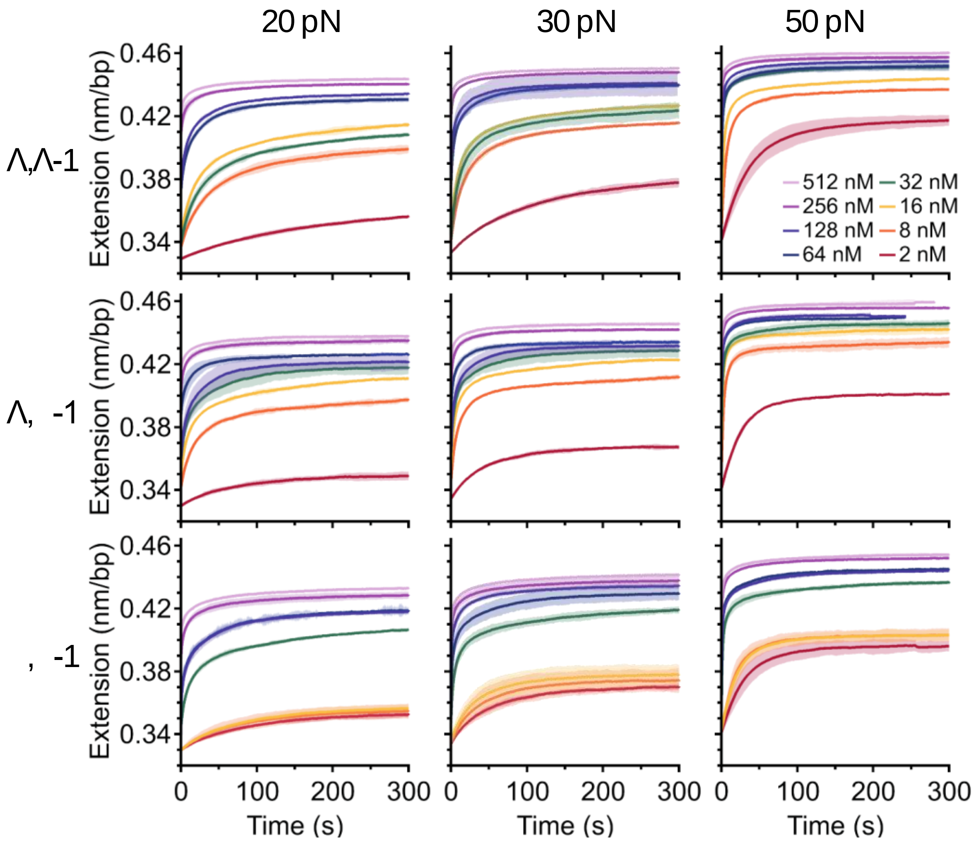

Supplementary Figure 1. Average extension-time trajectories Average extension-time trajectories for the three different stereoisomers at each force (20 – 50 pN) and concentration (2 – 512 nM). Shading shows standard deviation, n ≥ 3.

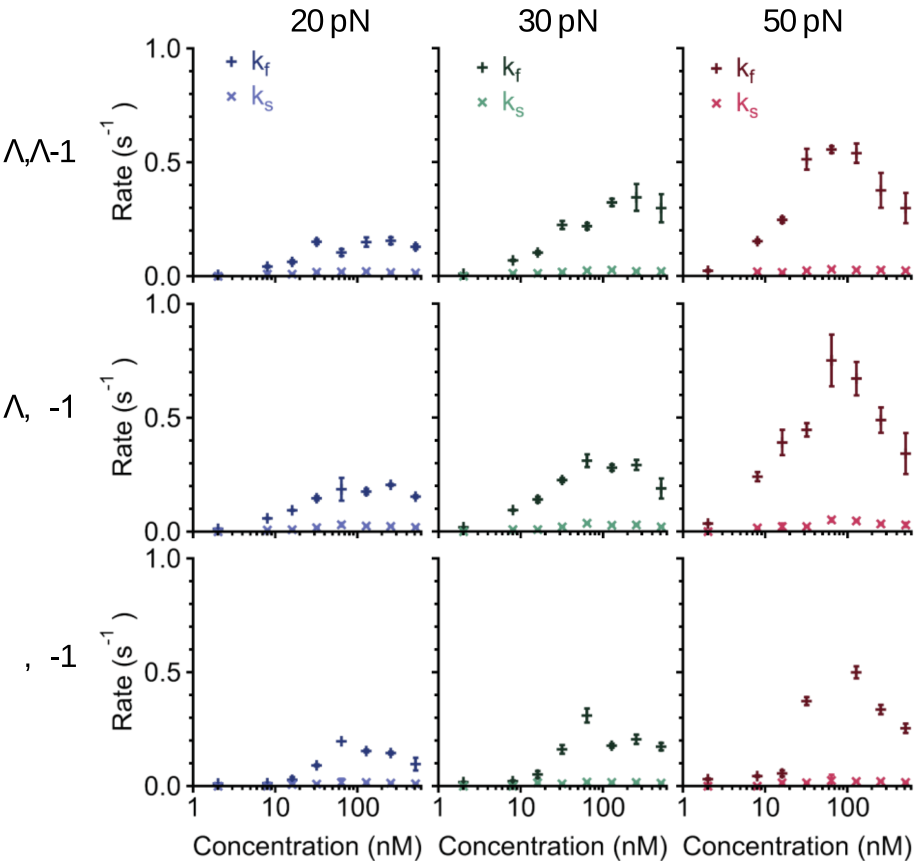

Supplementary Figure 2. Observed binding rates for each stereoisomer The observed fast, k_f, and slow, k_s, rate constants for DNA binding of Λ,Λ-1 , Λ,Δ-1 and Δ,Δ-1 at 20, 30 and 50 pN, obtained by fitting extension-time trajectories. The observed fast rate constant is concentration and force dependant. The observed slow rate constant is largely concentration and force independent. Error bars show standard deviation, n ≥ 3.

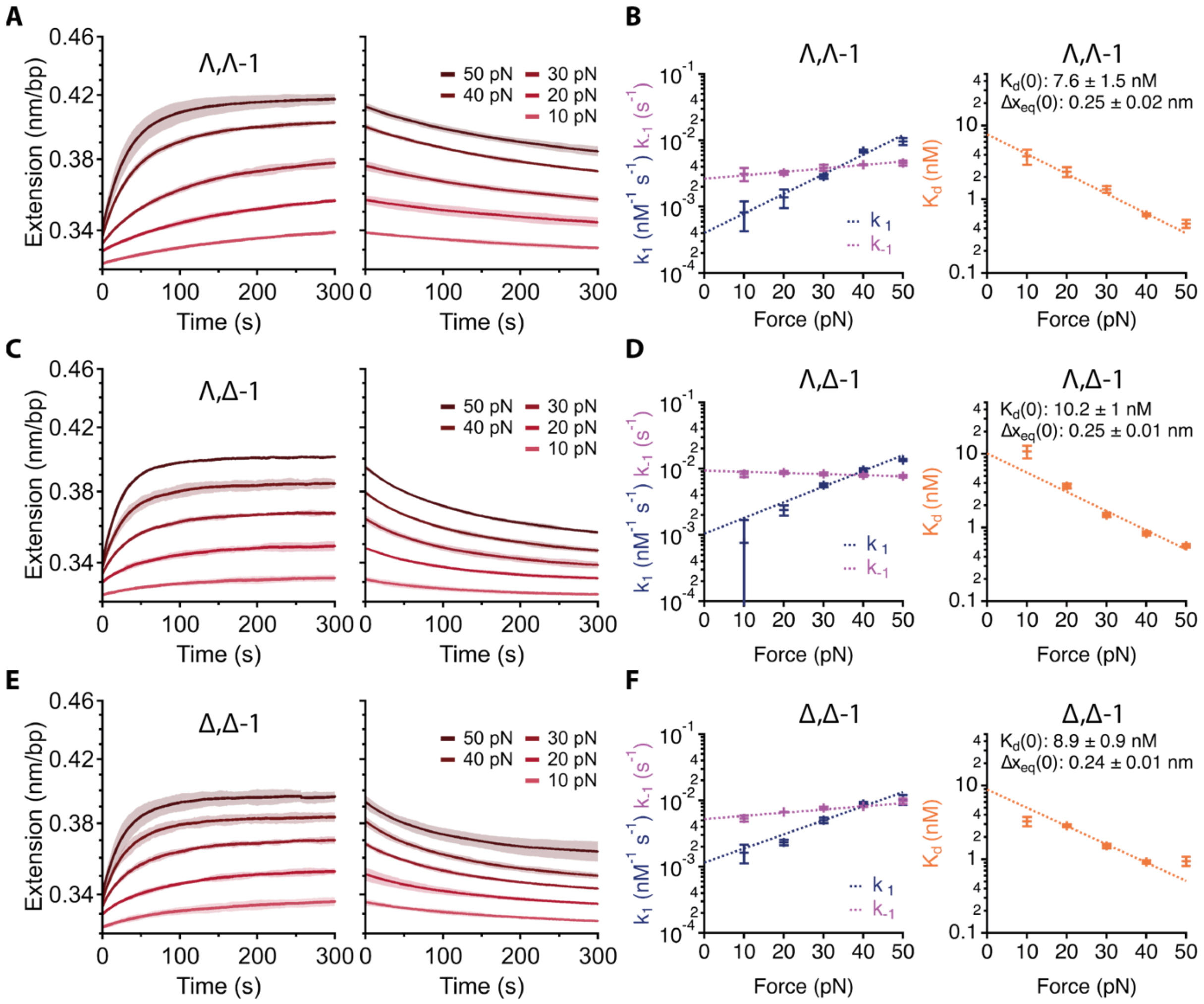

Supplementary Figure 3. Determining rate constants at low concentration (**A**) Average Λ,Λ-1 extension-time traces at 2 nM of association (left) and dissociation (right) for forces 10 – 50 pN. (n = 3, dark line = mean, light shaded = two standard deviations) (**B**) Force dependent elementary rate constants (left) and calculated equilibrium constants (right). Fit with exponential force dependance (Equation **5**) and extrapolated to give zero force values. (Error bars = s.d.) (**C,D**) Λ,Δ-1 average extension time traces and plots of elementary constants. (**E,F**) Δ,Δ-1 average extension time traces and plots of elementary constants.

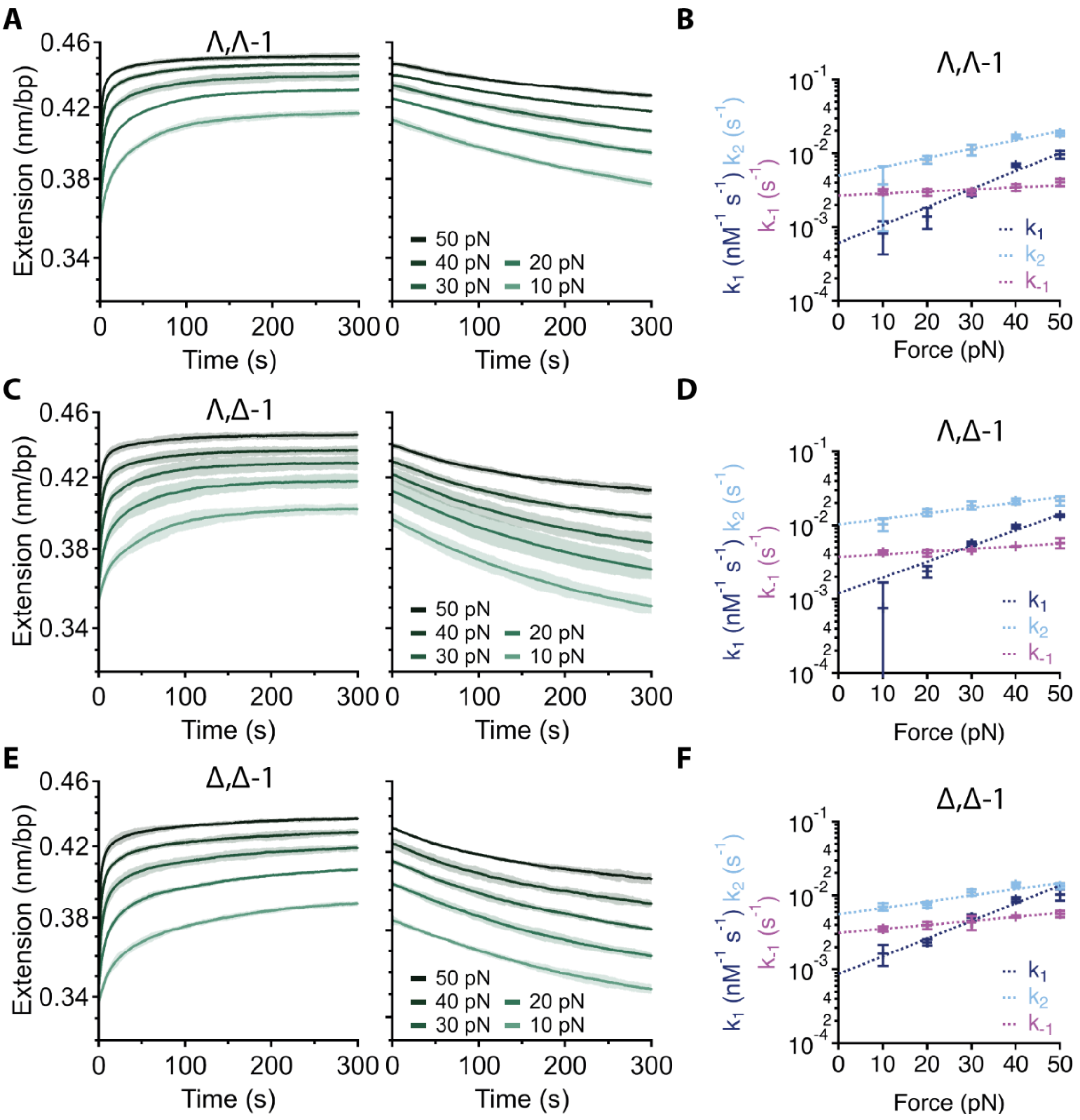

Supplementary Figure 4. Determining rate constants at high concentration (**A**) Average Λ,Λ-1 extension-time traces at 32 nM of association (left) and dissociation (right) for forces 10 – 50 pN. (n = 3, dark line = mean, light shaded = two standard deviations) (**B**) Force dependant elementary rate constants and observed dissociation rate (left). Fit with exponential force dependence (Equation **5**) and extrapolated to give zero force values. (**C,D**) Λ,Δ-1 average extension time traces and plots of elementary rate constants. (**E,F**) Δ,Δ-1 average extension time traces and plots of elementary rate constants.

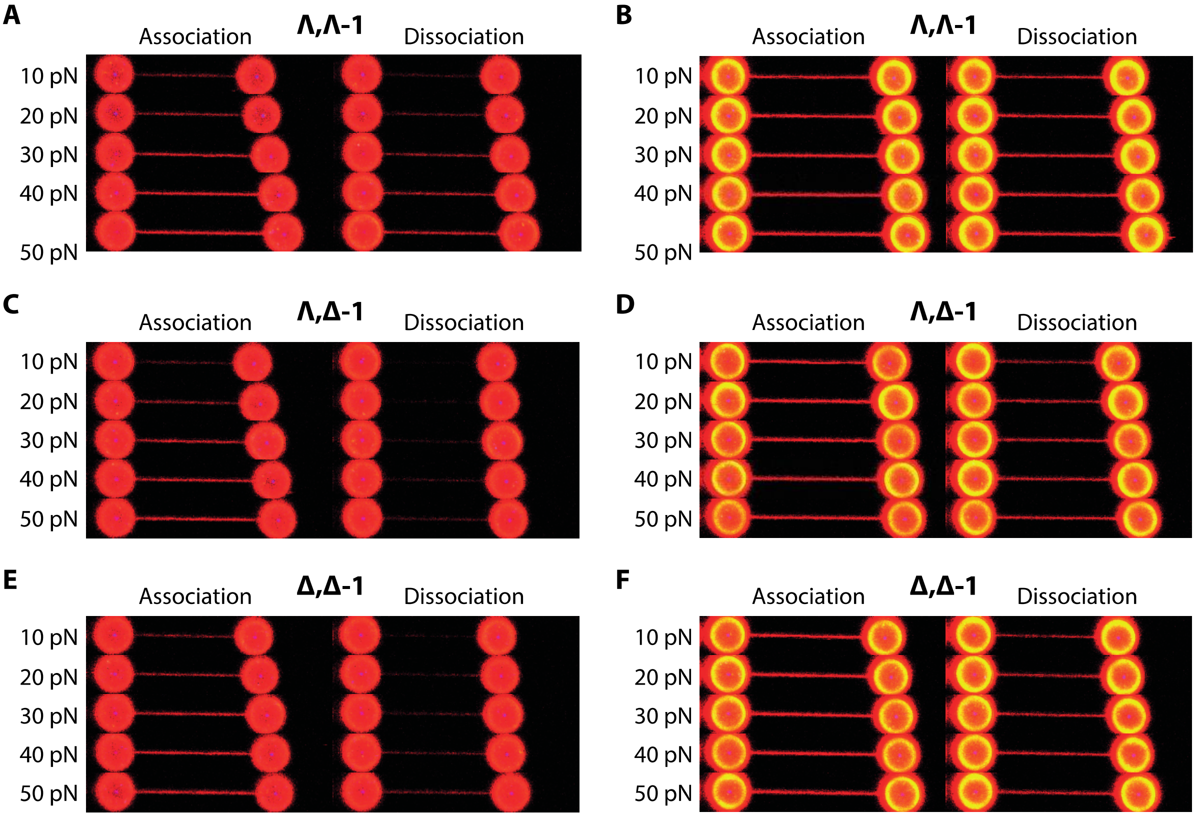

Supplementary Figure 5. **Comparison of fluorescence properties of stereoisomers.** (**A**) Confocal images of Λ,Λ-1 intercalator bound DNA held between the two trapped beads. The DNA was imaged after incubation with 2 nM Λ,Λ-1 under a constant force clamp (10 – 50 pN) for 5 minutes (“Association”, left). The same piece of DNA was then imaged after a further 5 mins held at constant force (10 - 50 pN) in the buffer only channel (“Dissociation”) (**B**) Confocal scans after ‘Association’ and ‘Dissociation’ with 32 nM Λ,Λ-1. (**C,D**) Λ,Δ-1 Confocal scans at 2 nM and 32 nM. (**E,F**) Δ,Δ-1 Confocal scans at 2 nM and 32 nM.

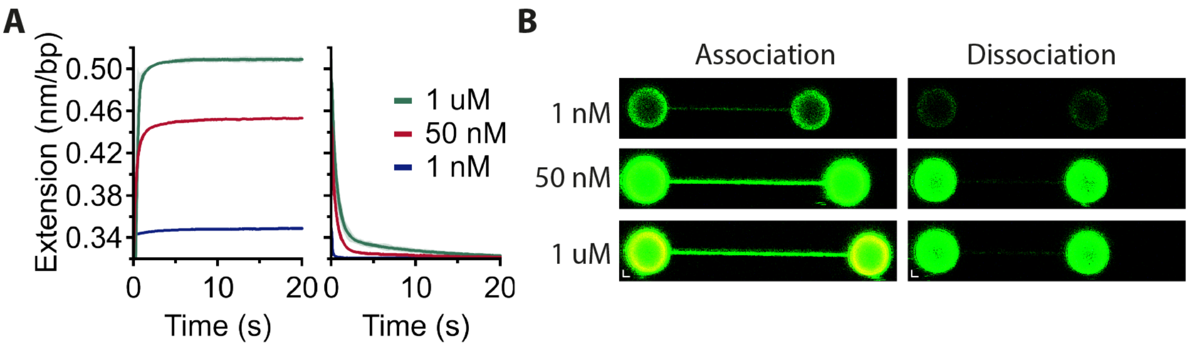

Supplementary Figure 6. **SYTOX binding and dissociation kinetics** (**A**) Average extension-time trajectories (n=3) for λ-DNA in the presence of different concentrations of SYTOX Orange for the “bind and lock” protocol. First the DNA is clamped at 50 pN for 1 minute (left). The DNA is then moved to buffer only channel and held at 5 pN for 1 minute (right). (**B**) Confocal images comparing the SYTOX emission at each concentration during the “bind and lock” protocol. A confocal scan is taken after a 1 minute 50 pN clamp (“Association”, left) and another scan after a 1 minute 5 pN clamp in the buffer only channel (“Dissociation”, right) Scale bar = 1 μm

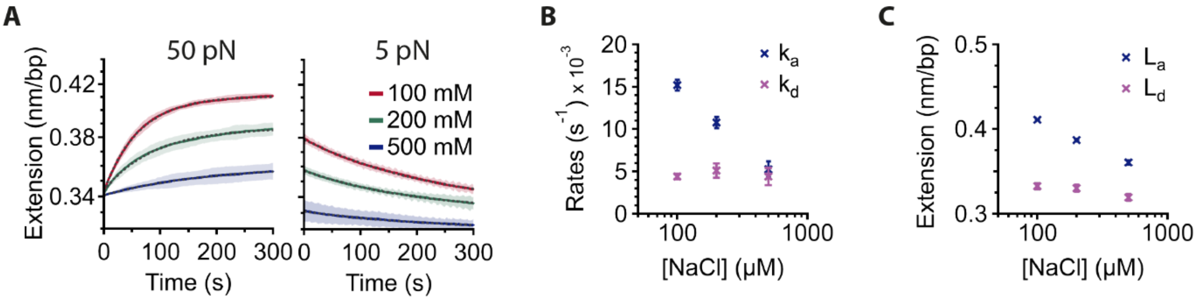

Supplementary Figure 7. **Effect of [NaCl] on Λ,Λ-1 binding kinetics** (**A**) Average extension-time trajectories (n=3) for λ-DNA in the presence of 1 nM Λ,Λ-1^{4+} in the "bind and lock" protocol in the presence of buffer containing different concentrations of NaCl. First the DNA is clamped at 50 pN for 5 minutes (left). The DNA is then moved to buffer only channel and held at 5 pN for 5 minutes (right). (**B**) Observed association and dissociation rates for Λ,Λ-1^{4+} in the "bind and lock" protocol with buffer containing 100 – 500 mM NaCl (**C**) Final extensions after association and dissociation for Λ,Λ-1^{4+} in the "bind and lock" protocol with buffer containing 100 – 500 mM NaCl

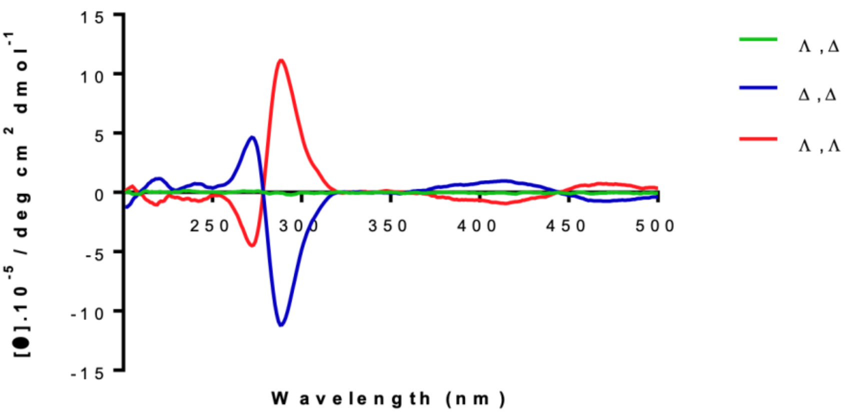

Supplementary Figure 8. Circular Dichroism spectra of the separated Λ,Λ-1^{4+}, Δ,Δ-1^{4+} and Λ,Δ-1^{4+} as a 15 µM solution in H_2O.

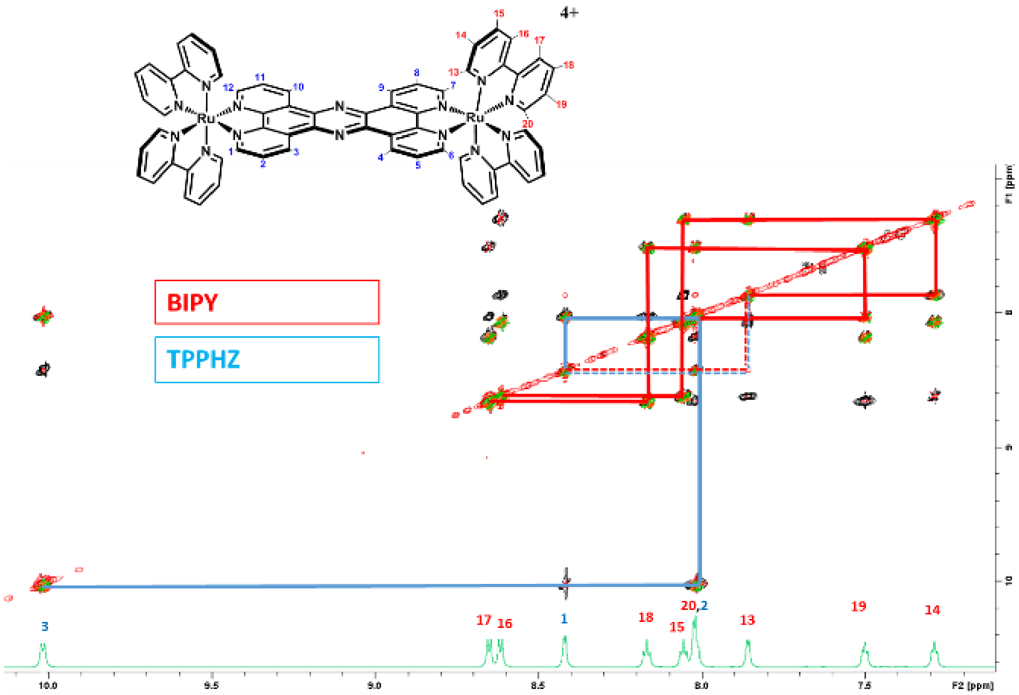

Supplementary Figure 9. 2D H1 NMR (top) and 1D H1 NMR (bottom) assignment of Λ,Λ-1^{4+}, Δ,Δ-1^{4+} and Λ,Δ-1^{4+} in D2O of free metal complexes, the NMR spectra are Identical for all three stereoisomers. 2D Spectra included; TOCSY (black), COSY (green) and NOESY (red). Blue and red lines correlate the spin systems of the tpphz and bipyridine, respectively. The dashed red/blue indicates a bipyridine-tpphz inter-ligand NOE cross peak.

Supplementary Data Tables

Supplementary Table 1. Calculated values of observed rate constant k_f at 32 nM concentration

Force (pN)	Observed k_f (s^{-1})		
	Λ,Λ-1^{4+}	Λ,Δ-1^{4+}	Δ,Δ-1^{4+}
10	0.03 ± 0.02	0.03 ± 0.02	0.06 ± 0.02
20	0.05 ± 0.03	0.08 ± 0.01	0.08 ± 0.01
30	0.10 ± 0.1	0.19 ± 0.01	0.17 ± 0.02
40	0.23 ± 0.3	0.31 ± 0.01	0.29 ± 0.03
50	0.36 ± 0.2	0.44 ± 0.02	0.34 ± 0.01

Supplementary Table 2. Rate constant k_s obtained from fitting at 32 nM concentration

Force (pN)	Observed k_s (s^{-1})		
	Λ,Λ-1^{4+}	Λ,Δ-1^{4+}	Δ,Δ-1^{4+}
10	0.004 ± 0.003	0.009 ± 0.002	0.006 ± 0.001
20	0.008 ± 0.001	0.014 ± 0.002	0.007 ± 0.001
30	0.011 ± 0.001	0.018 ± 0.002	0.011 ± 0.001
40	0.017 ± 0.001	0.021 ± 0.001	0.014 ± 0.001
50	0.018 ± 0.001	0.021 ± 0.002	0.013 ± 0.001

Supplementary Table 3. Sequences of End Caps used to make torsionally constrained λ-DNA. iBiodT = biotin modified T.

Name	Sequence
End Cap #1	`AGG TCG CCG CCC GGA GTT GAA CG/iBiodT/ /iBiodT/T/iBiodT/ T/iBiodT/A CGT TCA ACT CC`
End Cap #2	`GGG CGG CGA CCT CAA GTT GGA CAA /iBiodT/T/iBiodT/ T/iBiodT//iBiodT/ TGT CCA ACT TG`

Supplementary References

[1] P. Gutierrez-Escribano, M. D. Newton, A. Llauró, J. Huber, L. Tanasie, J. Davy, I. Aly, R. Aramayo, A. Montoya, H. Kramer, J. Stigler, D. S. Rueda, L. Aragon, *Sci. Adv.* **2019**, *5*, eaay6804.

[2] M. D. Newton, B. J. Taylor, R. P. C. Driessen, L. Roos, N. Cvetesic, S. Allyjaun, B. Lenhard, M. E. Cuomo, D. S. Rueda, *Nat. Struct. Mol. Biol.* **2019**, *26*, 185–192.

[3] O. Belan, C. Barroso, A. Kaczmarczyk, R. Anand, S. Federico, N. O'Reilly, M. D. Newton, E. Maeots, R. I. Enchev, E. Martinez-Perez, D. S. Rueda, S. J. Boulton, *Mol. Cell* **2021**, DOI 10.1016/j.molcel.2020.12.020.

[4] G. A. King, F. Burla, E. J. G. Peterman, G. J. L. Wuite, *Proc. Natl. Acad. Sci.* **2019**, *116*, 26534–26539.

[5] O. Belan, G. Moore, A. Kaczmarczyk, M. D. Newton, R. Anand, S. J. Boulton, D. S. Rueda, *STAR Protoc.* **2021**, *2*, 100588.
